# Characterization of a Chromosomal Type II Toxin–Antitoxin System *mazEaFa* in the Cyanobacterium *Anabaena* sp. PCC 7120

**DOI:** 10.1371/journal.pone.0056035

**Published:** 2013-02-25

**Authors:** Degang Ning, Yan Jiang, Zhaoying Liu, Qinggang Xu

**Affiliations:** 1 Department of Environment Sciences, School of the Environment, Jiangsu University, Zhenjiang, Jiangsu, China; 2 Institute of Life Sciences, Jiangsu University, Zhenjiang, Jiangsu, China; University of Padova, Italy

## Abstract

Cyanobacteria have evolved to survive stressful environmental changes by regulating growth, however, the underlying mechanism for this is obscure. The ability of chromosomal type II toxin-antitoxin (TA) systems to modulate growth or cell death has been documented in a variety of prokaryotes. A chromosomal *mazEaFa* locus of *Anabaena* sp. PCC 7120 has been predicted as a putative *mazEF* TA system. Here we demonstrate that *mazEaFa* form a bicistronic operon that is co-transcribed under normal growth conditions. Overproduction of MazFa induced *Anabaena* growth arrest which could be neutralized by co-expression of MazEa. MazFa also inhibited the growth of *Escherichia coli* cells, and this effect could be overcome by simultaneous or subsequent expression of MazEa via formation of the MazEa-MazFa complex *in vivo*, further confirming the nature of the *mazEaFa* locus as a type II TA system. Interestingly, like most TA systems, deletion of *mazEaFa* had no effect on the growth of *Anabaena* during the tested stresses. Our data suggest that *mazEaFa*, or together with other chromosomal type II TA systems, may promote cells to cope with particular stresses by inducing reversible growth arrest of *Anabaena*.

## Introduction

Cyanobacteria are an ancient and diverse group of prokaryotes found in many different habitats [1], which reflects the considerable ability of cyanobacteria to adapt to variable or extreme environmental factors such as nutrient availability, light, temperature [2]. Indeed, in their long evolutionary history, cyanobacteria have developed highly refined response strategies including both those shared with other prokaryotes during various stresses and those specific for individual species as a result of a particular stress, such as heterocyst formation of *Anabaena* sp. during nitrogen limitation. One common successful survival strategy is the ability to undergo reversible growth arrest observed in almost all prokaryotes [3], [4]. The roles of chromosomal toxin-antitoxin (TA) systems in regulation of bacterial growth is such a good example, which have been documented in many organisms, especially *Escherichia coli*, to cope with various stresses by reducing growth, inhibiting growth or killing a subpopulation of cells [5]–[8]. However, based on the authors' best knowledge there is no similar report in cyanobacteria so far.

TA systems, also referred to as addiction or suicide modules, typically comprise an auto-regulated operon encoding a stable toxin and a labile antitoxin [9]. Based on the antitoxin nature and mode of action, TA systems are grouped into type I, II and III classes. Antitoxins of type I and III systems are small RNAs that inhibit toxin expression (type I) [10], [11] or activity (type III) [12]. Antitoxins of type II systems are proteins that inactivate toxins by forming toxin-antitoxin (TA) complexes [9]. The identified TA toxins so far include several families with different biochemical activities [9], [13], [14]. The vast majority of type II toxins are mRNA specific endoribonucleases, also called mRNA interferases, such as MazF, RelE, HicA toxins [13]. Almost all described type II TA systems share mode of regulation: tight expression auto-regulation by a TA complex binding to the promoter region and toxin neutralization by formation of TA complex [9].

Type II TA systems were originally found in low copy number plasmids where they were first thought to be merely addiction modules to stabilize these systems. Recently, the discovery of their high prevalence on the chromosomes of free-living bacteria [15]–[17] led to the proposal that chromosomal type II TA systems are stress-response elements contributing to prokaryotes' adaptation to stressful environments [5], [9]. According to this hypothesis, under unfavorable conditions antitoxins would be degraded by stress-induced proteases, which causes relief of transcriptional repression of TA operons and release of toxins from TA complexes [9], [13]. As a consequence, the free toxins would induce reversible growth arrest or cell kill by inhibiting an essential cellular process, such as protein synthesis or DNA replication [5], [9]. This proposal has been supported by a series of recent experiment findings. For instance, in *E. coli*, activation of TA systems is triggered by various stresses [18]–[21], and ectopic expression of nearly all characterized chromosomal toxins could improve the ability to confer resistance to stress [22]–[24]. In addition, some TA knockout mutants have apparent phenotype changes under stress conditions tested. For example, a *mazEF*-deletion mutant exhibited the phenotype resistant to a series of stressful inductions including nutrient starvation, addition of antibiotics, DNA damage, oxidative and heat-shock stresses [19], [25], [26]. Therefore, by inducing a reversible growth arrest, TA toxins could allow stressed cells to remain in a dormant or non-growing stress-tolerant state until more favorable environmental conditions return.

The sequenced cyanobacteria genomes were also found to encode a large number of putative TA systems [15], [16]. However, it still remains to be investigated whether the homologs of the interesting TA systems of *E. coli* are as important in cyanobacteria. *Anabaena* sp. PCC 7120 (hereafter, *Anabaena*) is a filamentous heterocyst-forming cyanobacterium. This strain is amenable to genetic manipulation and has been used as a model to study multiple physiological processes in cyanobacteria. A comprehensive genome analysis revealed that the *Anabaena* chromosome possesses at least 38 putative TA loci belonging to various families of type II TA systems [16]. In this paper, we focus our study on characterization of a chromosomal gene pair *asl3212* and *all3211* (hereafter referred to as *mazEa* and *mazFa*, respectively) of *Anabaena*, which were predicted to constitute a sequence homolog of the best-studied *E. coli mazEF* system [15], [16]. This is the first functional characterization of TA *loci* in cyanobacteria.

## Materials and Methods

### Cyanobacterial strains, culture conditions and conjugations

Cells of *E. coli* strain DH5α or BL21(DE3) were grown in LB medium at 37°C with shaking at 200 rpm. When necessary, cultures were supplemented with ampicillin (Ap, 50 mg/l), spectinomycin (Sp, 100 mg/l) or kanamycin (Km, 50 mg/l). Cells of *Anabaena* strains were statically cultured in BG11 (containing 240 µM K_2_HPO_4_) medium [27] in 200-mL transparent plastic bottles at 30°C with a photosynthetic photon flux density of ∼30 µmol photons/m^2^.s, and shaken three times every day. As required, neomycin (50 mg/l) or spectinomycin (10 mg/l) was added. A series of modified BG11 media 1/10-P-BG11, 1/500-P-BG11, 1/1000-P-BG11 and P_0_-BG11 was prepared according to Schreiter [28] but simply modified here, which contain 24, 0.48, 0.24 and 0 µM K_2_HPO_4_, respectively. For the copper ion (Cu^+2^) -free cultures of *Anabaena*, deionized water with an electric resistance value of 18.2 MΩ was used for medium preparation, and cells of mid-logarithmic-phase cultures were harvested by centrifugation, washed three times with Cu^+2^-free BG11 (Cu_0_-BG11). The washed cells were re-suspended and cultured in Cu_0_-BG11 for at least 3-cycle passage to deplete intracellular Cu^+2^. Conjugations between *E. coli* and *Anabaena* were performed as previously described by Elhai [29].

### Construction of plasmids

Tool enzymes (TaKaRa, Dalian) were used following the manufacturer instructions. The primer sequences used are listed in Table S1. To facilitate the sub-clone of PCR-amplified products, appropriate restriction sites were added in some primers. The template for PCRs was the genomic DNA of *Anabaena* unless stated otherwise. All clones of PCR products were verified by DNA sequencing for subsequent experiments.

To create the plasmid for *mazEaFa* deletion, the left flank (LF) region of the *mazEaFa* operon was amplified by PCR using the primers mazEa-1 and mazEa-2, and ligated to pMD18-T (TaKaRa, Dalian) in the clockwise direction generating pJS134. The right flank (RF) region was amplified using the primers mazFa-1 and mazFa-2, and digested with *Xba*I/*Sac*I, and then cloned into the respective sites of pJS134 producing pJS141. The C.K2 cassette containing the kanamycin-resistance gene (Km^r^) was released from pRL446 [29] with *Xba*I, and ligated to the *Xba*I-digested pJS141 obtaining pJS153. The LF-C.K2-RF fragment was excised from pJS153 with *Bam*HI/*Sac*I and cloned in the *Bgl*II/*Sac*I sites of pRL277 [30], obtaining pJS167.

For copper-inducible expression plasmids, the fragment containing the promoter region (*P_petE_*) along with the start codon (ATG) of the gene *petE* (*all0258*) was amplified with the primers PpetE-1 and PpetE-2 and ligated to pMD-18T in the clockwise direction yielding pJS314. The *mazFa* gene was amplified using primer mazFa-Xb and mazFa-K, digested with *Xba*I/*Kpn*I and then cloned into the respective sites of pJS314 obtaining pJS334. The *mazEaFa* genes were amplified using the primers mazFa-Xb and mazFa-K, digested with *Xba*I/*Kpn*I and then cloned into the same sites of pJS314 generating pJS335. These recombinant fragments were individually released with *Pvu*II from pJS314, pJS334 and pJS335, and ligated to the *Pst*I/*Bgl*II, blunted with T4 DNA polymerase, sites of pHB912 [17], generating plasmids pJS350 (empty vector), pJS351 (containing *P_petE_-mazFa* fusion) and pJS352 (containing *P_petE_-mazEa-mazFa* fusion), respectively. These resulting shuttle plasmids can replicate in *Anabaena* cells.

To construct the plasmids for selective expression of MazEa and MazFa, the plasmid pJS298 was used, which contains *P_BAD_* and *P_T7_* as well as the genes *araC* and *lacI* encoding the regulator proteins of the respective promoters [31]. *mazFa* was amplified using the primers mazFa-N and mazFa-K, digested with *Nde*I/*Kpn*I, and cloned behind *P_T7_* of pJS298 generating pJS301. The *mazEa* gene was amplified using the primers mazEa-S and mazEa-K, and digested with *Sac*I/*Kpn*I, and then placed under *P_BAD_* of pJS301 obtaining pJS302.

The *mazEaFa* genes were amplified with the primers mazEa-N and mazFa-X and cloned into pMD18-T obtaining pJS792. This insert was released with *Nde*I/*Xho*I, and placed under *P_T7_* of pET30a (Novagen) resulting in pJS798, which allows the IPTG-inducible co-expression of MazEa together with MazFa tagged with 6 histidine (MazF-His_6_) at its C-terminus.

### Extraction of RNA and RT-PCR reaction

*Anabaena* cells were collected from 200 ml culture with 0.5–1.0 optical density at 730 nm (OD_730_) by centrifugation. The pellet of cells was used for RNA extraction as described previously [32]. Total RNA was converted to cDNA by reverse transcription using PrimeScript 1^st^ strand cDNA synthesis kit (TaKaRa, Dalian) according to the manufacturer's instructions. Using 1 µg of cDNA per reaction, the transcript abundance of *mazEa* and/or *mazFa* was determined by PCR amplification.

### Analysis of cyanobacterial growth rate

To analyze growth rate of the *Anabaena* strains, cells were grown in 100 mL antibiotic-free medium in 500 ml flasks in triplicate, and OD_730_ was measured at the selected time points. High-light exposure or phosphate limitation experiments were performed according to previous methods [33], [34] but simply modified here. To analyze the effects of high-light intensities, *Anabaena* cultures with an initial OD_730_ of 0.2 were grown under the light of 30, 100, 200 or 300 µmol photons/m^2^ s^1^. For phosphate limitation experiments, *Anabaena* cells were collected from the mid-logarithmic-phase cultures (OD_730_ of about 0.8), and washed twice with P_0_-BG11 medium. The cells were resuspended in 1/10-P-BG11, 1/500-P-BG11 and 1/1000-P-BG11, respectively, and further incubated under normal growth conditions. The induction by nitrogen deprivation was performed as previously described [32], and heterocyst formation of *Anabaena* cultures was investigated by microscopic analysis. To assess the effect of *mazEaFa*-encoded proteins, copper-free *Anabaena* cells harboring copper-inducible expression plasmids were cultured in BG11 containing 2 µM CuSO_4_ (Cu-BG11) at an initial OD_730_ of 0.2 under normal growth conditions.

### Assays of toxicity, antitoxicity and growth recovery

*E. coli* cells containing respective selection-expression plasmids were incubated in 100 ml of LB medium with glucose to an OD_600_ of about 0.6. For selective expression of the *mazEaFa* genes, 0.1 mM isopropyl β-D-thiogalactopyranoside (IPTG) or 0.2% (w/v) L-arabinose (Ara) was added for gene expression from the IPTG-inducible promoter *P_T7_* or the arabinose-inducible promoter *P_BAD_*, and 0.2% (w/v) glucose (Glu) was added to suppress the background expression from *P_T7_* and *P_BAD_*. For assays of cell growth on LB agar plates with corresponding inducers, 1 ml of each culture were serially diluted in 10-fold steps, and 2 µl of each of diluted samples was dropped on the agar plates with glucose, glucose and IPTG, glucose and L-arabinose or glucose, L-arabinose and IPTG, and then incubated for 30 h. For assays of rescue of *E. coli* growth arrest, each culture with an OD_600_ of 0.2 was divided into four equal parts, three of those were individually added with IPTG, L-arabinose or both, respectively. These cultures were further incubated as described above. Samples were taken every 30 min from each culture to measure OD_600_, and then the cells were harvested and washed three times with PBS buffer. The washed cells were resuspended in an equal volume of liquid LB medium, and 100 µl of appropriately diluted samples was spread on the plates containing glucose or along with L-arabinose. The colony-forming unit (CFU) was counted after incubation at 37°C for 30 h.

### Over-expression, purification and identification of MazEa and MazFa

Co-expression of MazEa with MazFa was induced by addition of 1 mM IPTG to the culture of *E. coli* BL21(DE3) containing pJS798 with an OD_600_ of ∼0.6, and growth was continued for 3 h. Co-purification of proteins was done by affinity chromatography using Ni^2+^-NTA His.Bind resins (Novagen, USA) under the native conditions, while purification of denatured proteins under the denaturing conditions according to the manufacturer's instructions. The purified products were separated with 15% SDS–PAGE. The densitometry of the bands purified under native conditions was then analyzed using Image J (http://rsb.info.nih.gov/nih-image/). The co-purified proteins were excised from gel, and determined by MALDI-TOF mass spectrometry (MS) analysis using a Shimadzu Biotech Axima TOF2 instrument (Shimadzu Biotech, Germany). The proteins embedded in gel slice was digested with trypsase and analyzed to determine the mass/charge (*m*/*z*) values according to the manufacturer instructions. The experimental peptide masses were compared to a theoretical cleavage with tyrpsin using the MS-DIGEST program (http://prospector.ucsf.edu).

## Results

### Genetic organization and transcriptional analysis of the *mazEaFa* operon

**Figure 1:**
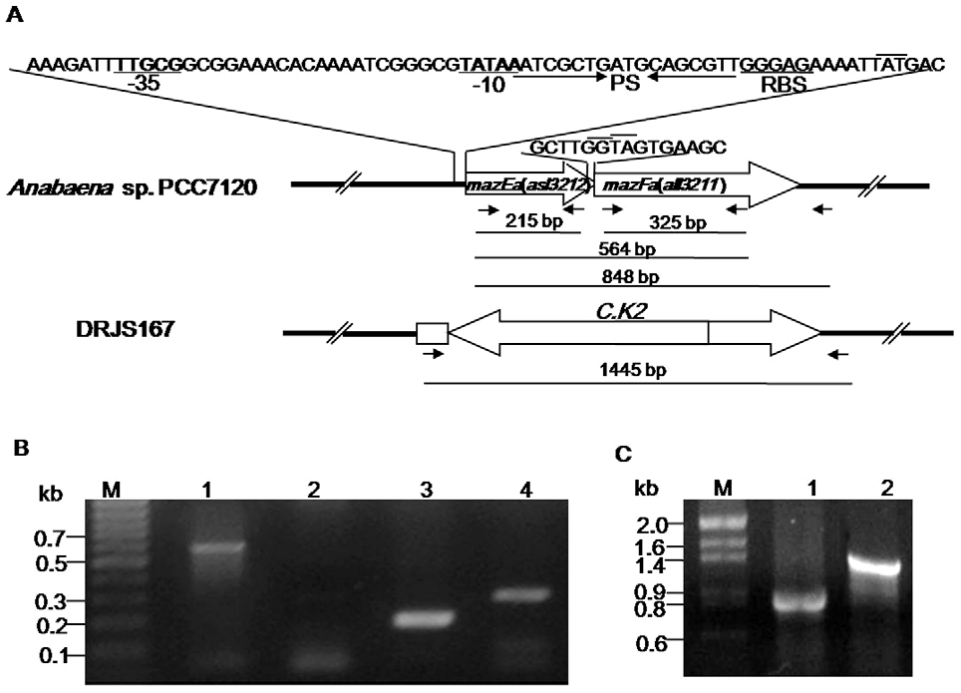
Genetic organization and allelic replace of the *mazEaFa* operon of *Anabaena*. (A) Schematic representation of the *mazEaFa* operon and its allelic exchange with C.K2 in the expected *mazEaFa*-deletion mutant. The ribosome binding site (RBS) and the palindrome sequence(PS)in the *P_mazEaFa_* promoter was underlined. The start and stop codons are overlined with short lines. Small arrows symbolize the relative location of the primers used for transcriptional analysis of *mazEaFa* in B and identification of the *mazEaFa*-deletion mutant in C, respectively. The PCR fragments and their expected sizes are shown below. (B) Detection of *mazEaFa* transcription by RT-PCR. The co-transcription of *mazEa* and *mazFa* was determined using the primers mazEa-R1 and MazFa-R2(lane 1) and the individual transcripts of *mazEa* (lane 3) and *mazFa* (lane4) using the primer pairs mazEa-R1/mazEa-R2 and mazFa-R1/mazFa-R2, respectively. RNA without reverse transcription served as a negative control (lane 2). All RT-PCR experiments were performed in triplicate, and consistent results were obtained. (C) PCR identification of allelic replacement of *mazEaFa* in a representative recombinant. The allelic exchange of *mazEaFa* with C.K2 was determined by PCR with the primers mazEa-R1 and MazFa-K, showing a band shift from 848 bp in the wild-type strain (lane 1) to 1445 bp in the DRJS167 strain (lane 2).

The *mazEa* (*asl3212*) and *mazFa* (*all3211*) genes on the *Anabaena* chromosome (http://www.kazusa.or.jp/cyano/) encode MazEa and MazFa homologous to the *E. coli* MazE (31% identity) and MazF (39% identity), respectively. MazEa is predicted to be an 80-residue protein with a molecular weight of 8.9 kDa and an isoelectric point of 4.94, while MazFa is predicted to be a 146-residue protein with a molecular weight of 16.5 kDa and an isoelectric point of 8.1. A genetic organization analysis revealed that *mazEa* is located upstream of *mazFa*, and the two genes overlap by one base pair (Figure 1A), apparently organized in a bicistronic operon. The inferred −35 (TTGCG) and −10 (TATAA) sites and the putative ribosome-binding sites (RBS, GGAGA) upstream of *mazEa* suggest that *mazEaFa* comprises a transcriptional unit with a putative σ70-family promoter (Figure 1A).

To characterize the coupling transcription between *mazEa* and *mazFa*, RT-PCR analysis was performed using primers that anneal to the 3′ end of *mazE*a and the 5′ end of *mazFa*. As seen in Figure 1B, the band with the expected size (564 bp) was observed after amplification of cDNA for *mazEaFa*, and confirmed by sequencing. The individual *mazEa* and *mazFa* genes were also amplified using the primers annealing to regions within the respective reading frames (Figure 1B). Possible DNA contamination can be excluded since no PCR products were evident in the negative controls (Figure 1B). These indicate that *mazEa* and *mazFa* are actively co-transcribed from a σ70-family promoter under normal growth conditions. Taken together, on the basis of the genetic organization and sequence homology of its products, the *mazEaFa* locus may constitute a putative *mazEF*-family TA system, where *mazFa* encodes for a toxin with bacterial mRNA interferases activity, and *mazEa* for the cognate antitoxin.

### Construction and characterization of mutant lacking *mazEaFa*

**Figure 2:**
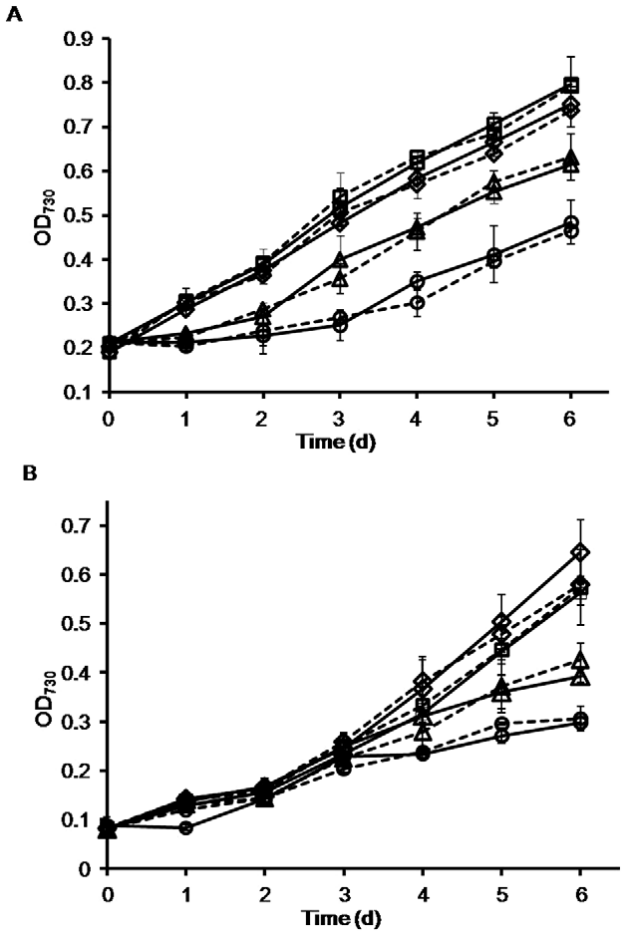
Effect of *mazEaFa* deletion on the growth of *Anabaena* strains during high-light exposure or phosphorus limitation. Growth profiles of the wild-type (solid lines) and *mazEaFa*-deletion (dot lines) strains of *Anabaena* under the light of 30 (diamonds), 100 (squares), 200 (triangles) and 300 (circles) µmol photons/m^2^ s^1^ (A), or in BG11 (diamonds), 1/10-P-BG11 (squares), 1/500-P-BG11 (triangles) and 1/1000-P-BG11 (circles) (B). Error bars indicate the standard error of the means from three independent experiments.

To investigate the physiological function of *mazEaFa*, the *mazEaFa*-deletion mutant was constructed by allelic exchange mutagenesis. The plasmid pJS167 was conjugally transferred into the *Anabaena* cells, the single-crossover and double-crossover recombinants were sequentially screened as previously described [30]. Twelve double-crossover recombinants were randomly selected and further cultured. As shown with PCR detection, *mazEa* and 5′ half of *mazFa* were replaced with C.K2 in 8 double recombinants, and the segregation was complete (Fig. 1C). Compared to their parental strain, the 8 double recombinants showed no any detectable difference in growth under the laboratory conditions (Figure 2), suggesting that the *mazEaFa* system is not essential for normal growth of *Anabaena*.

It has been documented that deletion of *E. coli mazEF* caused changes in response to a series of stresses, such as nutrient starvation, oxidative stress, antibiotic treatment, and DNA damage [19], [25], [26]. In nature, the adverse environmental factors cyanobacteria often encounter include high-light exposure and nutrient limitation, especially phosphorus or nitrogen starvation [2]. High-light exposure could reduce the photosynthetic electron transport components of cyanobacteria, resulting in excess production of reactive oxygen species (ROS) and eventually oxidative stress [35]. The nutrient limitation would inhibit growth of cyanobacteria [34], [36]. We thus examined whether the *mazEaFa* deletion could affect the growth of *Anabaena* under these major environmental stresses [35]. In addition, *Anabaena* is able to differentiate heterocysts, specialized nitrogen-fixing cells, at semi-regular intervals after nitrogen deprivation [37], effect of *mazEaFa* deletion on heterocyst development was also determined by microscopy. All these experiments were performed with three *mazEaFa*-deletion mutant strains, and similar results were obtained. As seen in Figure 2, under high-light exposure or phosphate limitation, both *mazEaFa*-deletion and wild-type strains showed no difference in growth rate. Furthermore, the mutant strains could develop mature heterocyst as observed in the wild-type strain after nitrogen starvation (Figure S1). These results demonstrate that, unlike the *E. coli mazEF*-deletion mutant, the *mazEaFa*-deletion mutant exhibits no discernible phenotype under the tested conditions. We randomly selected one of the *mazEaFa*-deletion strains for further experiments and designated it as DRJS167.

### Ectopic production MazFa induces growth arrest of *Anabaena*

**Figure 3:**
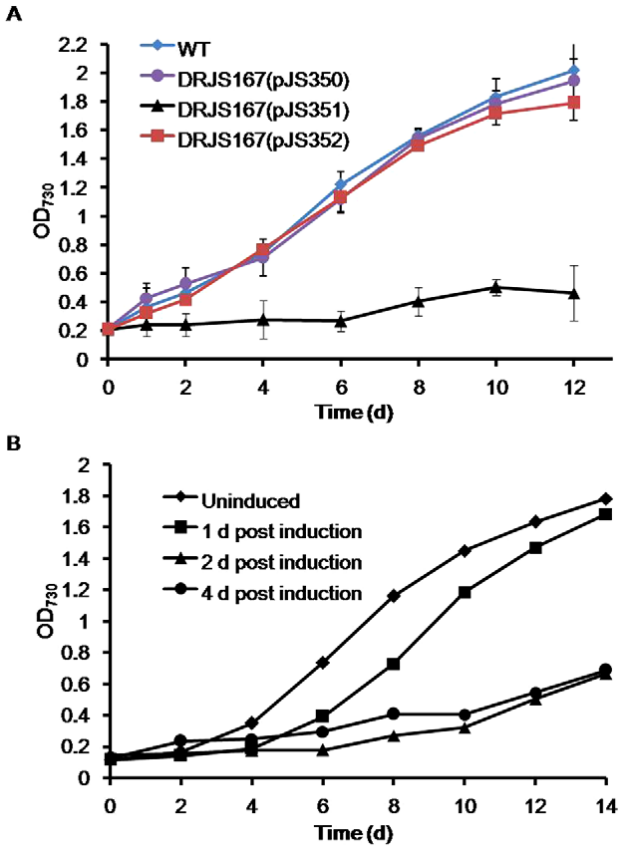
Effect of ectopic expression of MazFa or with MazEa on growth of *Anabaena*. Growth curves of the wild-type, DRJS167(pJS350), DRJS167(pJS351) and DRJS167(pJS352) strains cultured in Cu-BG11. The data are average of three replicate samples and the error bars represent standard error of the mean. (B) Representative experiments showing growth of DRJS167(pJS351) after the stop of MazFa production. Samples of DRJS167(pJS351) cultured in Cu^+2^-BG11 were taken at the indicated time points, and cells were collected and washed 3 times with Cu_0_-BG11. The pellets were re-suspended in the same medium for further incubation under normal conditions.

The crucial characteristic of TA systems is toxin-induced growth inhibition when in excess of their cognate antitoxins, we thus investigated the effect of ectopic expression of the *mazEaFa* genes on growth of *Anabaena*. For this, the tight copper-responsive promoter *P_petE_* [38] was used to control expression of *mazFa* from the plasmid pJS351 or co-expression of *mazFa* with *mazEa* from pJS352 (Figure S2) in *Anabaena* cells. To eliminate the possibility that the plasmid-encoded MazFa is interfered with the chromosome-encoded MazEa in the wild-type strain, pJS351 and pJS352 were conjugally transferred into the mutant DRJS167, generating strains DRJS167(pJS351) and DRJS167(pJS352), respectively. The strain DRJS167 (pJS350) (containing the empty vector) was used as a control. In the Cu_0_-BG11 medium, neither DRJS167(pJS351) nor DRJS167(pJS352) showed any noticeable difference in the growth rate relative to the control strain (data not shown). However, in the Cu-BG11 medium, DRJS167(pJS352) exhibited the growth rate similar to the control strain and the wild-type strain of *Anabaena*, while DRJS167(pJS351) displayed significant growth inhibition (Figure 3A). These indicate that the Cu^+2^-inudced ectopic expression of *mazFa* alone led to growth inhibition of *Anabaena*, but the co-expression of *mazEa* abolished this growth-inhibition effect, thus validating that MazFa functions as a toxin while MazEa is an antitoxin against MazFa.

Previous studies of TA systems showed that growth-inhibition effect of some toxins could be abolished by stopping the toxin synthesis in the absence of the cognate antitoxins [22], [39], [40]. We thus determined whether the MazFa-induced growth inhibition of *Anabaena* would be overcome only by subsequent stop of MazFa production via removal of Cu^+2^. As seen in Figure 3B, the cells which had been incubated for 1 d in the Cu-BG11 medium began to recover growth after 4 d of incubation in the Cu_0_-BG11 medium, while the cells incubated in Cu-BG11 for more than 2 d remained in an inhibited state under the same conditions. These suggest that growth inhibition of *Anabaena* by a prolonged induction (at least 2 d) with MazFa could not be relieved by stopping its production.

### MazFa expression leads to growth inhibition of *E. coli* that can be rescued by subsequent expression of MazEa

**Figure 4:**
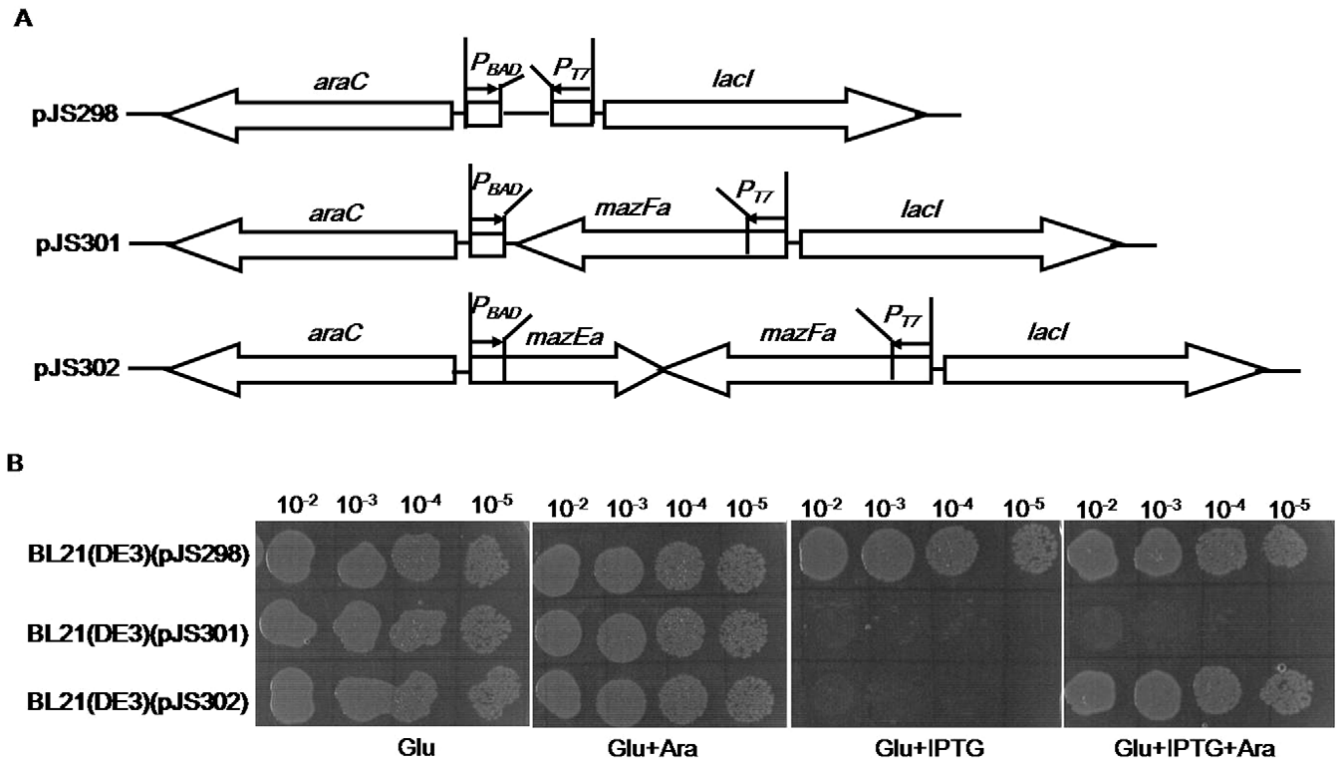
Effect of MazEa and/or MazFa production on growth of *E. coli*. (A) Schematic representation of plasmids for selective expression of *mazEa* and *mazFa* under control of P*_BAD_* and *P_T7_*, respectively. (B) Representative experiments showing growth of *E. coli* strains containing corresponding selection-expression plasmids on the agar plates with the respective inducers. Diluted samples of each culture were dropped on the plates containing glucose only (Glu), glucose and arabinose (Glu+Ara), glucose and IPTG (Glu+IPTG) or glucose, arabinose and IPTG (Glu+Ara+IPTG). Drop growth experiments were performed in triplicate, and similar results were obtained.

**Figure 5:**
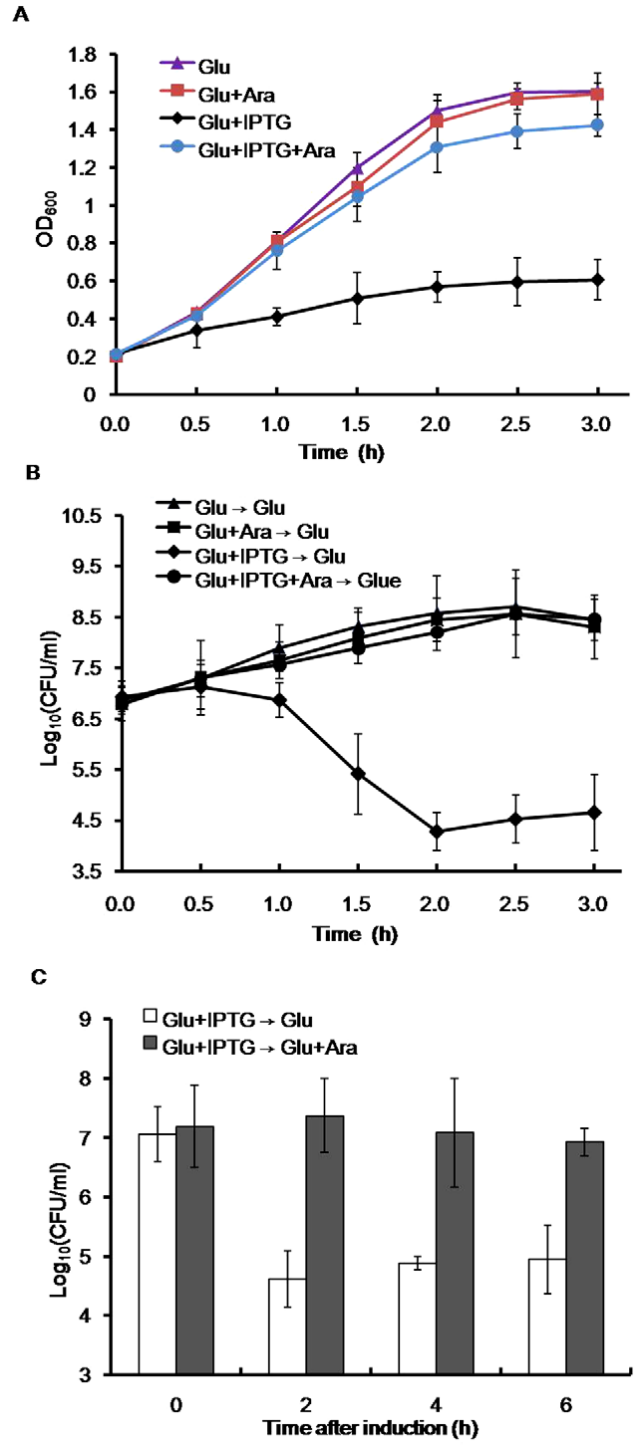
Rescue of MazFa-induced *E. coli* growth arrest. (A) Growth curves of BL21(DE3)(pJS302) in the liquid LB media containing the indicated inducers. (B) CFU counts of BL21(DE3)(pJS302) after the stop of production of MazFa and/or MazEa. Cells of BL21(DE3)(pJS302) cultures as seen in (A) were transferred at the indicated time points to the LB plates containing only glucose, and CFUs were counted after incubation. (C) Effect of subsequent production of MazEa on viability of the cells previously induced with MazFa. The IPTG-induced cells of BL21(DE3)(pJS302) were transferred at the indicated time points to the LB plates containing only glucose or with arabinose, and CFU counts were measured after incubation. Error bars indicate the standard error of the means from three independent experiments with three replicate samples.

In some characterized TA systems, the toxin toxicity could be counteracted only by subsequent production of antitoxin [41], [42], we next tested whether later expression of MazEa may inhibit the growth-inhibition effect of MazFa. *E. coli* has been successfully used as a host for verification of heterogenic TA components due to its elaborate conditional expression systems established [43]–[48], we thus investigated rescue effect of MazEa using the selection-expression system constructed here. This expression system includes *E. coli* BL21(DE3) cells harboring the selection-expression plasmids (Figure 4A). In these plasmids, the IPTG-inducible promoter *P_T7_* and arabinose-inducible promoter *P_BAD_* individually control the expression of *mazEaFa* genes *in trans*. So the strain BL21(DE3)(pJS301) can express MazFa in the presence of IPTG but not express MazEa under all circumstances. However, BL21(DE3)(pJS302) can express MazFa and/or MazEa upon induction with IPTG and/or arabinose. We first tested the effect of MazFa and/or MazEa production on *E. coli* growth on LB agar plates containing the respective inducers. As seen in Figure 4B, the presence of IPTG and/or arabinose has no effect on growth of BL21(DE3)(pJS298). However, BL21(DE3)(pJS301) could grow in the presence of arabinose alone but not in the presence of IPTG or along with L-arabinose. BL21(DE3)(pJS302) could grow on the plate containing arabinose or together with IPTG but not on the plate with IPTG alone. When these strains were cultured in liquid media containing IPTG, arabinose or both, the growth profiles of BL21(DE3)(pJS301) (data not shown) or BL21(DE3) pJS302 (Figure 5A) were similar to those observed on the plates with the corresponding inducers. These data indicate that the expression of *mazFa* alone led to growth inhibition of *E. coli*, and the co-expression of *mazEa in trans* abolished this growth-inhibition effect.

We next investigated whether stop of MazFa production in the absence of MazEa can overcome MazFa-induced growth arrest of *E. coli*. For this, expression of the *mazEaFa* components was selectively induced in BL21(DE3)(pJS302) with the respective inducers (Figure 5A), and subsequently switched off at the indicated time points by plating the cells on the plates without any inducer. As seen in Figure 5B, the earlier production of MazFa alone led to a reduction of about 10^3^ in CFU by 2 h of induction compared to the CFU at the time point of zero, whereas expression of MazEa or with MazEa showed no effect on CFU relative to the cells non-induced. Additionally, although a subpopulation of cells (2–5×10^−3^) exhibited colony-forming ability after the stop of MazFa synthesis, they were non-inheritable tolerant to MazFa because the similar reduction in CFU was observed when the cells from these colonies were re-plated on the plates with glucose (data not shown). This result, consistent with observation in *Anabaena* cells (Figure 3B), indicates that MazFa production caused inability of most *E. coli* cells to replicate even after the stop of MazFa synthesis in the absence of MazEa (Figure 5B). To examine whether subsequent expression of MazEa can reverse the MazFa-induced growth inhibition of *E. coli*, BL21(DE3)(pJS302) culture in the presence of IPTG alone, the CFU count was determined at time points of 0, 2, 4 and 6 h on the plates with or without arabinose. As seen in Figure 5C, the subsequent induction of MazEa increased CFU of the cells previously expressing MazFa at least to a level at the zero time point, but the absence of MazEa did not. These results indicate that MazEa could rescue the growth of almost all cells within at least 6 h.

### MazEa directly interacts with MazFa *in vivo*

**Figure 6:**
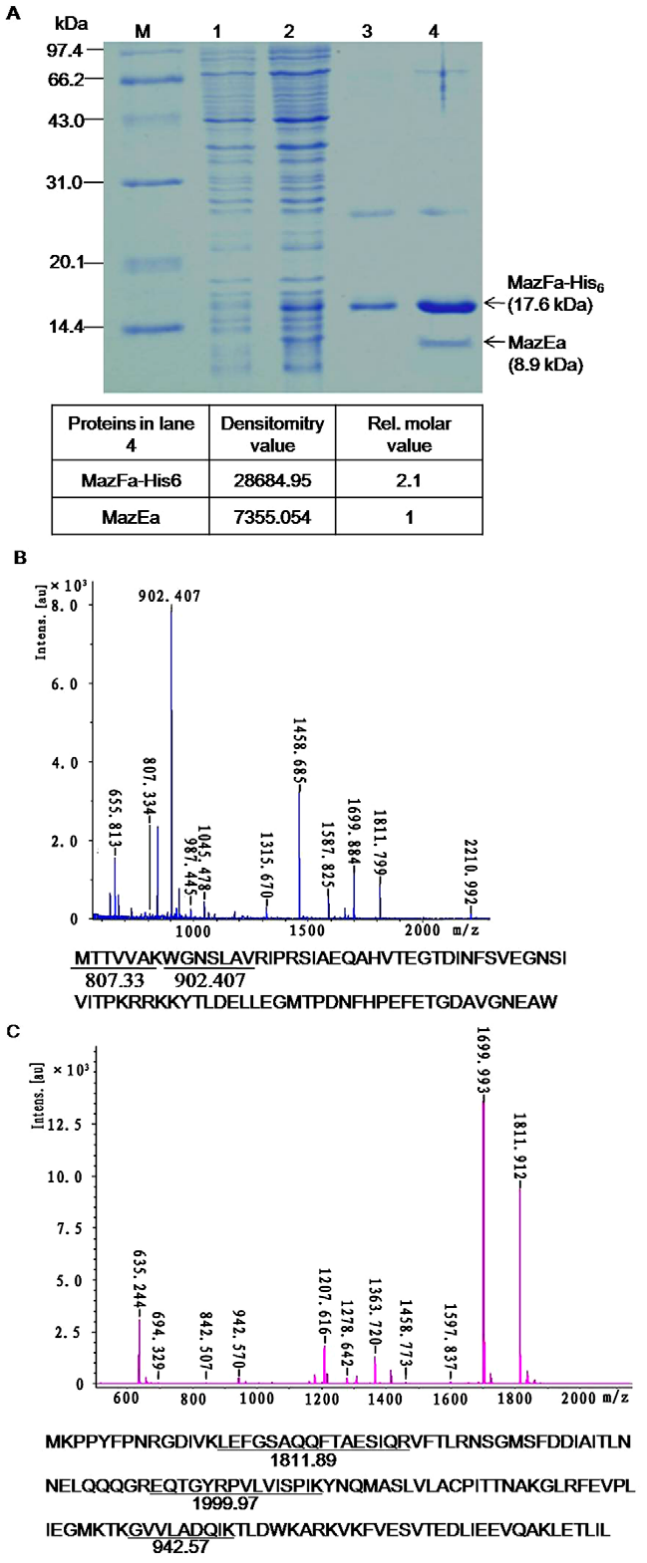
Interaction between MazEa and MazFa *in vivo*. (A) SDS-PAGE analysis of co-expression and purification of His_6_-MazFa and MazEa from BL21(DE3)(pJS798) cells. Lane 1, crude extract of un-induced cells; lane 2, crude extract of induced cells; lanes 3 and 4, products purified from induced cells under native and denaturing conditions, respectively; M, protein molecular weight standard with sizes as indicated in kDa. The bands indicated by arrows in lane 3 were subjected to mass spectrometer. The densitometry values and the relative molar ratio of MazEa to MazFa are shown below. (B) and (C) MS analysis of peptide mass fingerprinting of MazEa and MazFa-His_6_, respectively. Amino acid sequence and predicted m/z by online analysis of MazEa and MazFa are shown below.

To determine whether neutralization of the MazFa toxicity by MazEa is due to its interaction with MazFa to form the MazEa-MazFa complex *in vivo*, MazEa and MazFa-His_6_ were co-expressed in and co-purified from the strain *E. coli* BL21(DE3)(pJS798). As shown in Figure 6A, 9.9-kDa and 17.6-kDa proteins, consistent with the expected molecular masses of the recombinant MazEa and MazFa-His_6_ proteins, respectively, were co-expressed and successfully co-purified under native conditions (lane 3), but only the 17.6-kDa protein was purified under denaturing conditions (lane 4). MS analyses showed that 2 peaks (807.334 and 902.407 *m*/*z*) of the 8.9-kDa protein and three peaks (942.570,1699.993 and 1811.912 *m*/*z*) of the 17.6-kDa protein were found in the theoretical peak list of MazEa and MazFa (Figure 6B and C), respectively, in MS-DIGEST program. These data indicate that MazEa directly interacts with MazFa to form the MazEa-MazFa complex *in vivo*. A densitometrical analysis using Image J revealed that the relative molar ratio of MazEa to MazFa was 1∶2 (Figure 6A). Like many characterized TA complexes, the opposite pI values of MazEa (acidic) and MazFa (basic) may contribute to stabilization of the MazEa-MazFa complex [13].

## Discussion

In the present study, we confirmed that the chromosomal genes *mazEa* and *mazFa* of *Anabaena* form a bicistronic operon that is co-transcribed from its putative σ70-family promoter. *mazFa* encodes a toxic protein MazFa due to its growth-inhibition effect demonstrated in both cells of *Anabaena* and *E. coli*. This growth-inhibition effect of MazFa could be neutralized by MazEa through formation of MazEa-MazFa complex *in vivo*. Therefore, the *mazEaFa* locus has the properties required for a genuine TA system belonging to the *mazEF* family. By far, all of identified MazF homologues function as ribosome-independent endoribonucleases to effectively inhibit protein synthesis resulting in growth arrest, despite each with a different cleavage-site specificity among various species, such as ACA sequence for *E. coli* MazF [49] and GUUGC for *Myxococcus xanthus* MazF [50]. We thus reason that MazFa exerts its growth-inhibition effect via the mechanism of action similar to the identified MazF homologs.

Our studies also show that the MazFa-induced growth inhibition of *E. coli* could be completely abolished by subsequent production of MazEa at least within 6 h (Figure 5), suggesting bacteriostatic effect of MazFa. This result is in accordance with the previous demonstration that the overproduction of TA toxins caused reversible growth arrest [22], [39], [40], [42]. Therefore, it is reasonably proposed that the chromosomal *mazEaFa* system of *Anabaena* might represent a growth modulator that induces a reversible dormancy state to enhance fitness and competitiveness under particular stress conditions. As a result, cyanobacteria can persist a long period of time under stress conditions and revive when suitable conditions arise.

It is interesting to notice that growth analysis of the *mazEaFa*-deleted mutant under the selected stress conditions could not allow us to make an intimate connection between stress adaptation of *Anabaena* and *mazEaFa*. Indeed, by far only several TA knockout mutants have deleterious phenotypes, many TA deletion mutants show no discernible phenotype [5]. The most recent work demonstrated that progressive deletions of all ten TA systems encoding mRNAase toxins in *E. coli* have cumulative effect on formation of persister cells (a subpopulation of bacteria cells with low metabolic rates and antibiotic tolerance) [51], suggesting functional redundancy of TA systems. Comprehensive genome analyses also revealed the abundance and diversity of type II TA systems in cyanobacteria [15]–[17]. The unicellular cyanobacterium, *Synechocystis* sp. PCC 6803, contains as many as 34 putative TA systems [15]–[17], including our previously characterized *reNlEs* operon [31], [52]. Characterization of this TA system carried out in *E. coli* demonstrated that the *relNEs* operon constitutes a “hybrid” TA systems where the toxin gene *relEs* belonging to the *relE* family is paired with the antitoxin gene *relN* with no sequence homology to characterized antitoxin genes [52]. Furthermore, the antitoxin protein RelN in *E. coli* could be degraded by both Lons and ClpP2s proteases from *Synechocystis* sp. PCC 6803, thus resulting in the activation of the toxin RelEs from the RelN-RelEs complex [31]. *Anabaena* encodes at least 38 putative type II TA systems [15]–[17]. Perhaps because only one of these *Anabaena* TA genes was deleted here, the contribution of individual toxins might be marginal and detectable effects may require the cumulative action of multiple toxins *in vivo*.

Another reason for TA knockout mutants without deleterious phenotype may be due to our poor knowledge of the stressors activating TA systems of *Anabaena* cells. Various stresses have been documented to induce arrest of the cell cycle in cyanobacteria, as reported in other micro-organisms. For example, prolonged nitrogen starvation induced the cyanobacterium *Synechococcus* sp. PCC 7942 cells to enter a reversible growth arrest that represents a general acclimation process [36]. A long-term (at least 6 days) deprivation of phosphorus source also caused a reversible growth cessation of *Anabaena* sp. PCC 7120 [34]. Our data presented here show that the effect of *mazEaFa*-encoded products on growth of *Anabaena* or *E. coli* resembles the stress-induced reversible growth arrest. To determine a possible link of the TA system to a particular stress acclamation, we are investigating the conditions in which a particular toxin would be activated in the wild-type strain of *Anabaena* by promoter-reporter analysis.

Supposing their role in regulation of bacterial growth, chromosomal type II TA systems may promote cells to cope with various environment stresses. Therefore, the presence of a large number of type II TA systems in cyanobacteria may contribute to the impressive ability to acclimate variable environment conditions. For example, a bloom-forming cyanobacterium *Microcystis Aeruginosa NIES-843* contains as much as 97 putative TA systems, representing 1.5% with respect to the total number of predicted genes [17]. We thus speculate that such strikingly numerous TA systems might give the cyanobacteria cells a competitive advantage over other algae, resulting in cyanobacteria blooming under favorable environment conditions. Therefore, understanding of TA system-mediated growth regulation represents a promising avenue for clarifying the mechanism for environmental acclimation processes in cyanobacteria and developing novel and effective strategies to control blooms.

## Supporting Information

Figure S1 **Heterocyst formation of the** ***mazEaFa*** **-deletion mutant after starved of nitrogen.** The heterocysts of the wild-type and *mazEaFa*-deletion mutant strains of *Anabaena* were detected under a microscope before (+N) or after 24 h of induction (−N) by nitrogen deficiency. Arrowheads point to mature heterocysts.
(TIF)

Figure S2 **Schematic diagram showing the plasmids for the copper-inducible expression of MazFa or with MazEa.** The omega cassette is a spectinomycin (*Sp^r^*)/streptomycin(*Sm^r^*) resistance marker with stem loops at both ends that terminate background transcription. In the presence of copper, MazFa or together with MazEa was expressed from the copper-inducible promoter *P_petE_* in *Anabaena* cells.
(TIF)

Table S1 **Primers used in this study.**
(DOCX)
