# Supplementary figures and images for: Characterization of a Chromosomal Type II Toxin–Antitoxin System mazEaFa in the Cyanobacterium Anabaena sp. PCC 7120
Source: PLoS One. 2013 Feb 25;8(2):e56035. doi: 10.1371/journal.pone.0056035 (PMC3581536; doi:10.1371/journal.pone.0056035)

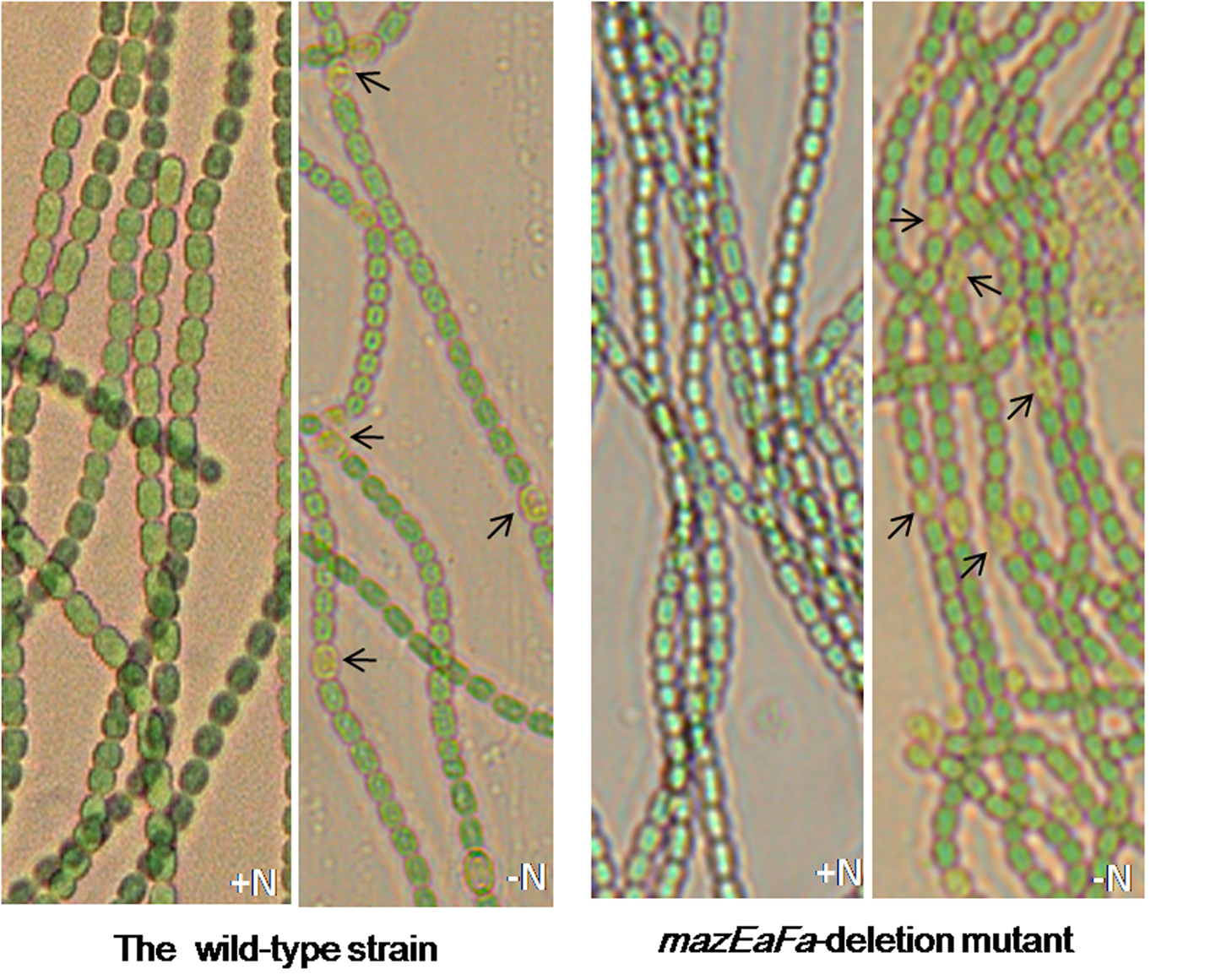

Supplement: Figure S1 — Heterocyst formation of the mazEaFa -deletion mutant after starved of nitrogen. The heterocysts of the wild-type and mazEaFa-deletion mutant strains of Anabaena were detected under a microscope before (+N) or after 24 h of induction (−N) by nitrogen deficiency. Arrowheads point to mature heterocysts. (TIF) [file pone.0056035.s001.tif]
