# Supplementary material for: Characterization of a Chromosomal Type II Toxin–Antitoxin System mazEaFa in the Cyanobacterium Anabaena sp. PCC 7120
Source: PLoS One. 2013 Feb 25;8(2):e56035. doi: 10.1371/journal.pone.0056035 (PMC3581536; doi:10.1371/journal.pone.0056035)
Supplement: Table S1 — Primers used in this study. (DOCX) [file pone.0056035.s003.docx]

**Table S1. Primers used in this study**

| **Primers** | **Sequences (5′-3′)^a^** | | **Res Restriction enzyme sites** |
| --- | --- | --- | --- |
| mazEa-R1 | CAACAGTTGTAGCTAAATGG | |  |
| mazEa-R2 | CACCTGTTTCAAATTCTGGTAC | |  |
| mazFa-R1 | CTTACTTTCCCAATAGAGGAGAC |  | |
| mazFa-R2 | TGATCTGCTAACACAACCCC | |  |
| mazEa-1 | GGATCCGAGTAACGGGGAGAACCACC | | *Bam*HI |
| mazEa-2 | ccatttagctacaactgttgtc | |  |
| mazFa-1 | ggagatctagagttccccttattgaaggaatg | | *Xba*I |
| mazFa-2 | ggagagagctcgtcatgtcttttggaatactg | | *Sac*I |
| PpetE-1 | CAAAATCTACTACTAAAGCCTG | |  |
| PpetE-2 | GGAGAATCTAGACATGGCGTTCTCCTAACCTG | | *XbaI* |
| mazEa-Xb | GGAGATCTAGAACAACAGTTGTAGCTAAATGGGG | | *XbaI* |
| mazFa-Xb | ggagaTCTAGAAAGCCGCCTTACTTTCCC | | *XbaI* |
| mazE-S | ggagagagctcGACAACAGTTGTAGCTAAATGGGG | | *Sac*I |
| mazEa-N | GGAGACATATGACAACAGTTGTAGCTAAATGGGG | | *Nde*I |
| mazEa-K | ggagaggtaccagtaaggcggcttcactacc | | *Kpn*I |
| mazFa-N | ggagacataTGAAGCCGCCTTACTTTCCC | | *Nde*I |
| mazFa-K | ggagaggtaccaagttgtatccgggtaactg | | *Kpn*I |
| mazFa-X | CTCGAGAATTAATGTTTCGAGTTTTGCTTGTAC | | *Xho*I |

^a^The restriction sites added are underlined.
